# Regional aspects of long-term public sector psychiatric care in the Eastern Cape

**DOI:** 10.4102/sajpsychiatry.v23i0.992

**Published:** 2017-05-09

**Authors:** Kiran Sukeri

**Affiliations:** 1 Department of Psychiatry, Faculty of Health Sciences, Walter Sisulu University, South Africa

## Abstract

**Objectives:**

The objective of this research was to determine regional aspects (such as clinical, geographic and socio-demographic) influencing the use of public sector long-term psychiatric services in the Eastern Cape. This is important in improving service delivery, to assist policy developers with evidence-based research and in providing equitable and efficient resource utilisation.

**Methodology:**

A situational analysis of Tower Psychiatric Hospital and Psychosocial Rehabilitation Centre (TPHPRC) in the Eastern Cape was conducted. Patient administrative data were utilised to determine geographic origin, date and age at admission, gender and diagnosis as of December 2015. The number of admissions from each region for the years 2010–2015 was also extracted from these data.

**Results:**

As of December 2015, there were a total of 390 patients at TPHPRC. Of these, 87% were male patients. The average age at admission for male and female patients was 36 years and 44 years, respectively. Of the patients, 53% originated from the western regions and 57% of female patients presented with a dual diagnosis. The highest number of admissions was in 2015, with the majority originating from Port Elizabeth.

**Conclusion:**

Despite higher access to public psychiatric care in the western region, the majority of patients originated from there. Contributing factors to this include diagnoses, insufficient bed numbers and the absence of admission criteria and referral pathways. It is recommended that the provincial Department of Health set up a task team to determine a standardised working framework for all public sector psychiatric institutions. This should be informed by national policies, legislation and provincial norms and indicators.

## Introduction

The World Health Assembly adopted the Comprehensive Mental Health Action Plan 2013–2020 in May 2013.^1^ This plan has at its core to improve mental health in all 194 member states by 2020. It also focuses on the determinants of mental health such as economic and social factors. The plan sets out four objectives, and objective 2 refers to the provision of a comprehensive, integrated and responsive mental health and social services in community-based settings. In line with this, South Africa’s National Mental Health and Policy Framework^2^ aims to ensure that mental health services are equitable, comprehensive and integrated at all levels of the health system. It advocates a move away from long-term psychiatric care towards the development of community psychiatry within a 7-year period.

However, prior to rapid deinstitutionalisation, it would be important to investigate the current utilisation of long-term psychiatric care provincially, specifically, the origin, demographic profile and reasons associated for admission. This information will provide the foundation for the development of an accessible community psychiatric service, which is as close to where patients reside. It will also inform the development of adjunctive services, such as substance-abuse rehabilitation centres, ambulatory services and child and adolescent facilities.

There is a wide variation within the Eastern Cape Province in the access to and availability of mental health services. The western region is more urbanised, has easier territorial access to public sector psychiatric care and non-governmental organisations (NGOs) that provide residential and day care facilities than in the central and eastern areas of the province.^3^ The province has no child and adolescent services and only one public sector long-term psychiatric facility.

Tower Psychiatric Hospital and Psychosocial Rehabilitation Centre (TPHPRC), originally Fort Beaufort Asylum, has been in existence since 1894.^4^ TPHPRC is situated in Fort Beaufort in the Amathole District Municipality of the Eastern Cape. The hospital provides specialised psychiatric care in the form of medium- to long-term care and psychosocial rehabilitation services for the entire Eastern Cape Province. This translates to providing a public sector psychiatric service to an approximate population of 6.5 million.^5^ This is a 400-bed facility consisting of 344 male and 56 female beds. There are 2 female and 12 male wards, providing care for long-term patients, geriatric patients, intellectually impaired patients, sick and frail patients and reclassified state patients. The current staff component comprises 1 psychiatrist, 2 medical officers, 1 clinical psychologist, 2 occupational therapists, 5 social workers and a total of 175 nurses of various categories. Previous research in this institution focused on its history and staff and bed distribution in the province.^4,6^ This research paper focuses on regional aspects in the use of medium- to long-term public sector care at TPHPRC. There are no studies from this province that demonstrate regional variations in the utilisation of long-term public sector psychiatric services.

As on 31 December 2015, TPHPRC did not have admission guidelines. All admissions were governed solely by the *Mental Health Care Act* 17 of 2002 (MHCA).^7^ This process meant legal admissions were in effect only after the promulgation of the MHCA in 2004. Patients transferred were required to have all MHCA documents, including the MHCA 16 and a brief referral form completed. There was no requirement for reports from Social Workers, Clinical Psychologists or detailed clinical notes. Prior to 2004, patients were transferred irrespective of diagnosis and age.

Therefore, the objective of this research was to determine the factors influencing the utilisation of medium- to long-term psychiatric services in the Eastern Cape. This information would provide an evidence base in the commissioning and planning of public sector mental health services with particular reference to medium- to long-term care. It would also allow for the equitable and efficient delivery of mental health services.

## Methodology

The study was a situational analysis of medium- to long-term psychiatric service provision at TPHPRC, Eastern Cape. The study included all patients at the institution as of December 2015. The study was conducted as follows:

1. The patient administration officer at the institution was requested to provide a spreadsheet of all patients at the institution.
2. The information extracted from this spreadsheet was date of admission, gender, age at admission, current age, diagnosis and geographic origin. This was then grouped according to the five main locations where mental health facilities are available in the province, viz. Queenstown, Mthata, East London, Grahamstown and Port Elizabeth.
3. After grouping patients into their area of origin, the following descriptive statistics were calculated for male and female patients; minimum, maximum, mean, median, mode, standard deviation and standard error of the mean for age at admission. The same calculations were done for the total number of male and female patients. The online software, OpenEpi, developed by the Centers for Disease Control (United States) (available at: http://www.openepi.com), was utilised to calculate descriptive statistics.
4. Further information extracted included the number of admissions from each region to TPHPRC for the years 2011–2015.
5. For ease of analysis, the diagnoses were grouped as single, dual (mental disorder with either a co-occurring substance-related disorder *or* a general medical condition [GMC]) or triple (mental disorder with co-occurring substance-related disorder *and* a GMC). The data were analysed for the commonest GMC and substance of abuse. This was done per region of origin.

## Results

There was a total of 390 patients admitted to TPHPRC as on 31 December 2015, consisting of 337 male and 53 female patients. Approximately 55% of male patients and 40% of female patients originated from Port Elizabeth. There were no patients from the Queenstown area. Table 1 demonstrates the territorial distribution of these patients in the Eastern Cape.

**TABLE 1:** Regional distribution of patients at Tower Psychiatric Hospital and Psychosocial Rehabilitation Centre as on 31 December 2015.

Area	*N* (%)	Male patients	Female patients
Port Elizabeth	206 (53)	185	21
East London	88 (23)	76	12
Grahamstown	68 (17)	52	16
Mthata	20 (5)	17	3
Outside the province	8 (2)	7	1

**Total**	**390**	**337**	**53**

The mean age at admission for male and female patients was 36 and 43 years, respectively. The 5-year-old (male) and 9-year old (female) were children with intellectual disability.

The descriptive statistics for patients from each region are shown in Table 2. Male patients from all regions were younger on admission compared to female patients. The highest number of admissions originated from the Port Elizabeth and East London areas. The number of admissions from all areas except East London increased in 2015. The number of admissions from the Port Elizabeth area increased 2.5 times in 2015 compared to 2014, as demonstrated in Table 3.

**TABLE 2:** Descriptive statistics per region at admission to Tower Psychiatric Hospital and Psychosocial Rehabilitation Centre.

Variable	Port Elizabeth		East London		Grahamstown		Mthata		Outside the province	
				
	Male patients	Female patients	Male patients	Female patients	Male patients	Female patient	Male patients	Female patients	Male patients	Female patients
*N*	185	21	76	12	52	16	17	3	7	1
Minimum age (years)	7	11	9	9	7	17	5	35	21	0
Maximum age (years)	67	73	58	72	59	67	61	58	60	0
Mean (years)	35.93	41.33	38.5	42.17	36.44	46.25	33.06	43.60	36.71	0
Median (years)	34	43	40.5	43	38.5	46	34	0	37	0
s.d.	11.29	17.46	12.07	15.77	12.69	11.64	13.84	-	13.65	-

s.d., standard deviation.

**TABLE 3:** Number of admissions to Tower Psychiatric Hospital and Psychosocial Rehabilitation Centre for the years 2011–2015.

Area	2011	2012	2013	2014	2015
Port Elizabeth	22	8	25	26	64
East London	10	8	7	8	6
Grahamstown	7	0	6	10	11
Mthata	3	4	3	1	4
Outside the province	0	0	0	0	3

Of the female patients, 57% presented with a dual diagnosis, the majority originating from the East London area. The most frequent diagnosis was schizophrenia with a GMC, followed by intellectual disability with a GMC (Table 4). When the same population was analysed for GMCs, HIV ranked the highest followed by hypertension, non-insulin-dependent diabetes mellitus and asthma. In the triple-diagnosis category, all patients presented with co-occurring HIV. The commonest substance of abuse was cannabis followed by alcohol.

**TABLE 4:** Diagnosis of female patients on admission to Tower Psychiatric Hospital and Psychosocial Rehabilitation Centre.

Diagnosis	% per diagnosis per region					

	Port Elizabeth	East London	Mthata	Grahamstown	Outside the province	Total
Single	29%	25%	67%	44%	100%	36%
	50%: Schizophrenia	67%: Schizophrenia	50%: Schizophrenia	43%: Schizophrenia	100%: Schizophrenia	
	33%: ID	33%: ID	50%: NCD	29%: Bipolar mood disorder type 1		
	17%: Bipolar mood disorder type 1			14%: ID		
Dual	57%	67%	33%	14%: Epilepsy	0%	57%
	33%: ID + GMC	25%: Schizophrenia + GMC*	100%: Schizophrenia + GMC			
	25%: Schizophrenia + GMC	25%: Bipolar mood disorder type 1 + GMC				
	25%: NCD + GMC	13%: Bipolar mood disorder type 1 + SUD				
	17%: Bipolar mood disorder type 1 + GMC	13%: ID + GMC				
		13%: Schizophrenia + ID				
Triple	14%	8%	0%	0%	0%	8%
	66%: Schizophrenia + SUD + GMC	100%: SUD + HAND¹ + GMC				
	34%: Bipolar mood disorder type 1 + SUD + GMC					

GMC, general medical condition; ID, intellectual disability, NCD, neurocognitive disorder; SUD, substance-use disorder; 1 = HIV-associated neurocognitive disorder.

Among the male population, the majority (53%) presented with a single diagnosis, schizophrenia being predominant. The most frequent medical diagnosis was epilepsy, followed by hypertension, asthma and HIV. Overall cannabis alone, cannabis in combination with methaqualone (white pipe) and alcohol were the commonest substances abused. Among the male population from Port Elizabeth, Crystal methamphetamine (Tik) featured as the third commonest substance of abuse after cannabis and white pipe. The triple-diagnosis male patients from both Port Elizabeth and East London presented with cannabis as the most frequent substance of abuse. The co-occurring GMC in Port Elizabeth group was hypertension, while in the East London group it was HIV (Table 5).

**TABLE 5:** Diagnosis of male patients at admission to Tower Psychiatric Hospital and Psychosocial Rehabilitation Centre.

Diagnosis	% per diagnosis per region					

	Port Elizabeth	East London	Mthata	Grahamstown	Outside the province	Total
Single	45%	62%	59%	64%	57%	53%
	69%: Schizophrenia	60%: Schizophrenia	60%: Schizophrenia	73%: Schizophrenia	100%: Schizophrenia	
	17%: ID	15%: ID	20%: ID	12%: ID		
	7%: Bipolar mood disorder type 1	13%: Bipolar mood disorder type 1	20%: Bipolar mood disorder type 1	6%: Bipolar mood disorder type 1		
	4%: Schizoaffective disorder (bipolar type)	9%: Epilepsy		6%: SUD		
	3%: Psychosis not otherwise specified	2%: NCD		3%: Epilepsy		
	1%: Epilepsy					
Dual	50%	37%	41%	37%	43%	44%
	63%: Schizophrenia + SUD	32%: Schizophrenia + SUD	57%: Schizophrenia + GMC	37%: Schizophrenia + SUD	67%: Schizophrenia + SUD	
	17%: Schizophrenia + GMC	25%: Schizophrenia + GMC	29%: ID + GMC	26%: Schizophrenia + GMC	33%: ID + GMC	
	7%: ID + GMC	18%: ID + GMC	14%: Major depressive disorder + GMC	21%: ID + GMC		
	5%: Bipolar mood disorder type 1 + SUD	11%: Bipolar mood disorder type 1 + GMC		10%: Bipolar mood disorder type 1 + GMC		
	3%: NCD + GMC	7%: ID + SUD		5%: Bipolar mood disorder type 1 + SUD		
	1%: Bipolar mood disorder type 1 + GMC	7%: NCD + GMC				
	1%: ASPD + SUD					
	1%: SUD** + GMC					
	1%: ID + ADHD					
Triple	5%	1%	0%	0%	0%	3%
	100%: Schizophrenia +	100%: Schizophrenia +				
	SUD + GMC	SUD + GMC				

ID, intellectual disability; SUD, substance-use disorder; GMC, general medical condition; ASPD, anti-social personality disorder; NCD, neurocognitive disorder; ADHD, attention-deficit hyperactivity disorder.

## Discussion

The results of this research show a higher number of admissions from the western compared to the eastern regions of the province, despite a higher access to public sector mental health services in the western region.^6^ There is a plethora of factors that could have singularly or in combination contributed to this phenomenon. These include socio-economic factors, facility-specific factors, diagnosis, comorbidities, lack of family support, inappropriate behaviours, territorial accessibility and governance.

Socio-economic factors such as unemployment status, income and education levels may have impacted on the rate of utilisation of mental health services. There is a definite association between poverty and mental illness.^3,8^ Studies have demonstrated that prior to diagnosis, persons with schizophrenia resided in areas of higher social deprivation, therefore, suggesting that social decline began during the prodromal period of the illness.^9^ People with the lowest socio-economic status have an eight times higher risk for schizophrenia than those in the highest socio-economic status.^10^ In the Eastern Cape, the South African Index for Multiple Deprivation^11^ shows that the wards most deprived in terms of material possessions, social and human capital, decent housing, basic services and poverty are in the eastern regions, particularly in the former Transkei, and the least deprived wards are centred around the two nodes of economic development, viz. Port Elizabeth and East London. This implies that a higher utilisation of mental health services would be expected from the eastern compared to the western region; however, results from this study do not correlate to this theory.

Therefore, there are other social and health factors that are driving the increased use of long-term services from the western region. Increased urban migration and the development of informal settlements around these economic hubs could be contributing factors. The majority of households living in shacks in the Eastern Cape are concentrated around these two urban centres.^12^ Access to water and electricity supply, sanitation and refuse removal is poor in these informal settlements; there are higher rates of unemployment than formal residential areas and the average income is ZAR R800/month, thus indicating a significantly high level of deprivation.^13^ Research has shown that although psychiatric conditions may occur at higher rates in poorer communities, they may also cluster together in disintegrating urban communities.^14^ This means that infra-territorial differences may not be accounted for in the development of deprivation indices and the high rate of mental health service utilisation in these two urban nodes may be driven by the populace of informal settlements. More research is required on accessibility to health services and utilisation rates by this population group in urban centres.

A facility-specific factor is the lack of capacity to accommodate patients in listed public sector mental health services, which could then drive the increased need for the deviation of admissions to long-term care. In the western region of the province, patients are initially assessed at Dora Nginza Hospital for 72 h then transferred to Elizabeth Donkin Hospital (EDH). EDH has capacity for 163 beds; however, their admissions exceed this capacity. The excess patients are accommodated on floor beds, at an average of 22 per month (personal communication with the Head of Department, EDH: February 2016). All alternative care facilities, such as ‘step-down’ or community residential homes, are provided by NGOs and/or private facilities,^3^ making access difficult in terms of payment for services and the limited number of beds in these sectors.

The high comorbidity of substance abuse among male patients was a contributing factor to the high rate of admissions to public sector facilities and ultimately an increased use of long-term care. According to the Medicine’s Research Council Report for 2015,^15^ the western region has access to five NGOs, one private hospital and two public sector facilities that provide substance-abuse rehabilitation services. One public sector facility is a 38-bedded unit for minors only and the other is based at Fort England Hospital, Grahamstown and accepts adults. Because of the limited number of beds, the majority of patients are dependent on NGOs, which accept state-funded patients.^15^ The cost of a 21-day in-patient programme at one centre is approximately R28000.00.^15^ Despite this high cost, the NGO sector in the western region admitted 1180 patients as of December 2014, of whom approximately 70% were male patients, 70% of the total admissions were treated as in-patients and none were state funded.^15^ This same report demonstrates that the commonest substances of abuse among the general male population is similar to that in the male psychiatric population of the western region.

These two factors indicate the need for increased inter-sectoral collaboration between the public and non-governmental sectors, in improving the placement of patients and the utilisation of alternative care structures. Improved co-ordination between the social and health sectors has demonstrated a decrease in the utilisation of long-term hospitalisation.^16^

Lyketsos et al.^17^ have reported that medical comorbidity can impact on mental health outcomes and functionality and increase the length of stay; therefore, addressing the medical condition during acute in-patient admission may directly influence the length of stay and improve psychiatric outcomes thus avoiding long-term care. In this study, the majority of patients presented with a passive medical comorbidity (defined as a condition that after admission did not need referral to another medical specialist for modification of treatment either because it was existent and known to the patient prior to admission or it was discovered during admission and was pharmacologically controlled by the attending psychiatrist),^18^ such as hypertension, asthma and non-insulin–dependent diabetes. The high rate of HIV among female patients is comparative to that of long-stay patients at Weskoppies Hospital, Pretoria.^19^ At the East London Mental Health Unit, the average length of stay for female patients was calculated at 28 days,^20^ which is higher than the recommended national target norm of 17 days.^21^ This could be because of the high incidence of medical comorbidities, as reported by Uys^22^ in 2013, when the HIV prevalence rate among female in-patients was 13%. These medical conditions should not directly impact on the requirement for long-term psychiatric care.

Social work reports from TPHPRC (unpublished data) indicate a consistent lack of support from family members, particularly from the western regions. Therefore, long-term psychiatric admission becomes a solution for these patients. The medical model of management needs to be inclusive of family in the treatment process. This increased involvement provides the opportunity for education and advocacy and the development of prevention strategies.

Across all regions of the province, there is an over-representation of male patients between the ages 30 and 45 years (average age 36 years). This high rate of male admissions could be attributed to the behavioural disorders associated with psychosis that results in patients being treated in psychiatry units only, as reported by Benetier.^23^ This phenomenon is not exclusive to the Eastern Cape or the Republic.^24,25^ These difficult to discharge patients are then transferred to long-term institutions for further care.

A specific governance factor was the release of Circular 1/2/1/2 of 2015^26^ by the provincial Department of Health, which could explain the increased number of admissions from the western regions in 2015.The purpose of this circular was ‘to improve the quality of admission of mental health patients in the province’; however, it contains contradictory information. While in the initial sentence it requires all hospitals to conform to the requirements of the MHCA^7^ and its regulations, it also directed all specialised institutions to accept all patients irrespective of the correct MHCA^7^ forms or the hospital being full.

The low number of admissions from Mthata, a poorly resourced centre in terms of public sector mental health services could be because of several factors. These could include a high unmet need because of the absence of clear referral pathways and the lack of public and private sector mental health resources. There are no community residential facilities, either public or NGO provided.^3^ This is a vast geographic region with limited in-patient and ambulatory services, thus indicating the absence of screening for mental disorders at the primary and secondary healthcare level. There could also be a lack of perception of care for mental illness as compared to physical conditions among the general population. Another contributing factor could relate to territorial accessibility, which refers to the distance between a service and its user’s place of residence. TPHPRC is located approximately 318 km away from Mthata. To this must be added journey time and the cost of travel for family members. This implies that patients are treated away from their communities rather than within their own supportive environments, which would ensure recovery. There is clearly a need for increased research in this region to determine factors relating to public sector psychiatric service utilisation.

## Conclusion

It is recommended that the increased provision of in-patient and ambulatory services in the western region will improve early detection of mental illness and hence early initiation of appropriate management. There is also a clear need for the development of public sector services for substance-abuse disorders and increased inter-sectoral collaboration. An upscaling of mental health service provision is required in the eastern regions. Previous research in the province outlines evidence-based strategies to achieve this.^3,6,20,27^

This research clearly demonstrates an inequitable public sector mental health service. There are two main principles^28^ that should govern policy development: improving access to evidence-based care and respecting the human rights of affected persons. Regional access is an integral element of equity in healthcare.

The unilateral decision of Circular 1/2/1/2^26^ demonstrates the lack of political commitment to find a common solution in line with the principles set out by the National Mental Health Plan.^4^

It is recommended that the provincial Department of Health set up a task team with all the relevant stakeholders to determine a standardised working framework for public sector psychiatric institutions. This should be informed by national policies, legislation and provincial norms and indicators.
